# Preparation of Bifunctional Orthosilicophosphate MgO‐CaO‐ZnO‐P_2_O_5_‐SiO_2_ Glasses: In Vitro Evaluation of Antibacterial Activity and Osteoblast Gene Expression Behavior

**DOI:** 10.1002/adhm.202502546

**Published:** 2025-09-06

**Authors:** Sungho Lee, Hayato Asano, Makoto Sakurai, Takayoshi Nakano, Toshihiro Kasuga

**Affiliations:** ^1^ National Institute of Advanced Industrial Science and Technology (AIST) Nagoya 463–8560 Japan; ^2^ Department of Applied Chemistry College of Engineering Chubu University Kasugai 487–8501 Japan; ^3^ Division of Materials and Manufacturing Science Graduate School of Engineering The University of Osaka Osaka 565–0871 Japan; ^4^ Division of Advanced Ceramics, Graduate School of Engineering Nagoya Institute of Technology Nagoya 466–8555 Japan

**Keywords:** antibacterial activity, bioactive glasses, biomaterials, cell behavior, dissolution behavior, osteocalcin, structure

## Abstract

Phosphate and phosphate invert glasses contain various elements, with a wide range of compositions. Recently, our group reported orthosilicophosphate glasses (SPGs) and the glass network structure composed of orthophosphates and orthosilicates crosslinked by cations. ZnO is an intermediate oxide that improves the chemical durability of glass. Additionally, Zn^2+^ ions exhibit antibacterial activity and stimulate bone formation. In this work, ZnO‐containing SPGs are prepared for biomedical applications. The glasses are mainly composed of PO_4_, SiO_4_, MgO_4_, and ZnO_4_ orthotetrahedral structures. The ZnO‐containing SPGs exhibit excellent antibacterial activity, with bacterial counts > 5 orders of magnitude lower than that of the control. Meanwhile, ZnO‐containing SPGs have mild inhibitory effects on cell proliferation by Zn^2+^ ions; however, they exhibit significant upregulation of osteogenic markers compared with the control owing to the release of inorganic ions from the glasses. The ZnO‐containing SPGs prepared in this work exhibit bifunctional properties suitable for biomedical applications. They serve as bioadaptive materials capable of controlling gene expression by releasing therapeutic ions.

## Graphical abstract

Bifunctional ZnO‐containing orthosilicophosphate glasses (SPGs) composed mainly of orthotetrahedral groups with the unique glass network structure without long‐chain units are prepared via melt‐quenching. The glasses exhibit excellent antibacterial activity and upregulate the expression of osteogenic markers by releasing inorganic ions. They can be used as bioadaptive materials to control gene expression.

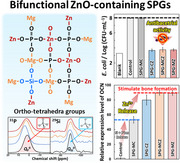

## 1 Introduction

Phosphate glasses have low melting temperatures and high acidity, and they can readily incorporate various inorganic ions at different concentrations.^[^ ^1^ ^]^ In addition, they can be vitrified under relatively low content of network formers (NWFs), such as phosphate.^[^ ^1^ ^]^ However, phosphate glasses are highly reactive with aqueous solutions, and their chemical durability must be controlled because of acidification due to excess dissolution for biomedical applications.^[^ ^2^ ^]^ In our previous work, phosphate invert glasses (PIGs) were prepared by adding intermediate oxides such as TiO_2_ and Nb_2_O_5_ to improve the chemical durability and degree of glassification.^[^ ^3^, ^4^ ^]^ These intermediate oxides can function as NWFs and network modifiers (NWMs) in glass network structures.^[^ ^5^ ^]^ Invert glasses contain <40 mol% NWFs and are predominantly composed of pyrophosphate (*Q*_P_^1^) and orthophosphate (*Q*_P_^0^).^[^ ^6^, ^7^, ^8^ ^]^ PIGs are composed of short phosphate groups, such as *Q*_P_^0^ and *Q*_P_^1^. The glass network structure exhibits a highly ionic bonding state, with *Q*_P_^0^ and *Q*_P_^1^ bonded via nonbridging oxygen coordinated by modifier ions.^[^ ^3^, ^9^ ^]^ Therefore, PIGs can contain various inorganic ions (e.g., therapeutic ions), and their dissolution behavior can be controlled by tailoring their glass network structure.^[^ ^10^, ^11^ ^]^ PIGs are expected to stimulate bone regeneration by releasing therapeutic ions.^[^ ^12^, ^13^ ^]^

Recently, our group reported the preparation of orthosilicophosphate glasses (SPGs) using a melt‐quenching method.^[^ ^14^, ^15^ ^]^ In most SiO_2_‐P_2_O_5_ glass systems, metasilicate or metaphosphate networks contain orthophosphate or orthosilicate, respectively.^[^ ^16^, ^17^, ^18^ ^]^ Phase separation occurs in silicate and phosphate networks.^[^ ^16^ ^]^ SPG has a unique glass network structure composed of *Q*_P_^0^ and orthosilicate (*Q*_Si_^0^), indicating that it does not contain long‐chain structures. SPGs contain MgO, which is classified as an intermediate oxide^[^ ^5^ ^],^ and crosslinking of the phosphate and silicate tetrahedra improves the glass‐forming ability. However, SPGs also contain P─O─Ca and Si─O─Mg bonds, which are easily hydrolyzed in aqueous solutions and exhibit an excellent ion‐release ability compared with PIGs.^[^ ^14^ ^]^

Inorganic ions (e.g., ions showing therapeutic properties) released from glasses stimulate various cellular functions.^[^ ^19^ ^]^ Silicate and Ca^2+^ ions released from Bioglass 45S5 stimulate osteoblast proliferation by increasing the production of insulin‐like growth factor II^[^ ^20^ ^]^ and upregulate alkaline phosphatase (ALP) and osteocalcin (OCN) for osteoblast differentiation.^[^ ^21^ ^]^ Phosphate ions are essential for the regeneration of mineral tissue in bone. Additionally, they upregulate the expression of matrix Gla protein, which is a key regulator of bone formation.^[^ ^22^ ^]^ The Mg^2+^ ion is an important element in the human body, and its concentration in the body is strongly related to bone strength.^[^ ^23^ ^]^ Additionally, these ions stimulate osteoblast adhesion,^[^ ^24^ ^]^ proliferation,^[^ ^25^ ^]^ differentiation,^[^ ^26^ ^]^ and calcification.^[^ ^25^ ^]^

ZnO can be classified as an intermediate oxide^[^ ^27^ ^],^ and in glass, it can function as either NWF or NWM.^[^ ^28^ ^]^ Moreover, ZnO can improve the chemical durability and glassification degree of PIGs by forming P─O─Zn bonds.^[^ ^29^ ^]^ In contrast, Zn^2+^ ions exhibit antibacterial properties^[^ ^30^, ^31^ ^]^ and have inhibitory effects on human dental plaque.^[^ ^32^ ^]^ In the case of cell functions, Zn^2+^ ions, which are essential trace elements^[^ ^33^ ^]^ upregulate ALP, osteopontin (OPN), and OCN^[^ ^34^, ^35^, ^36^ ^]^ and enhance bone formation by promoting Runx2‐targeted osteoblast differentiation gene transcription.^[^ ^37^ ^]^ Furthermore, Zn^2+^ ions inhibit osteoclastic bone resorption by suppressing osteoclast formation and activity.^[^ ^33^, ^38^ ^]^

In this work, ZnO‐containing SPGs with various ZnO contents were investigated to simultaneously enhance bone regeneration and antibacterial properties. MgO‐CaO‐ZnO‐P_2_O_5_‐SiO_2_ SPGs were prepared by substituting CaO/MgO with ZnO and evaluating the changes in glass structure. Their ion‐release behavior was measured in a Tris‐HCl buffer solution (TBS), and their antibacterial properties were evaluated using gram‐negative (*E. coli*) and gram‐positive (*S. aureus*) bacteria. The gene expression levels of osteoblasts cultured with medium for ion extraction from the glasses were analyzed for osteogenic markers to assess their potential for biomedical applications.

## 2 Results

### 2.1 Preparation of MgO‐CaO‐ZnO‐P_2_O_5_‐SiO_2_ Glasses

**Table** 1 presents the compositions of the glasses. The nominal and analyzed compositions exhibited no significant differences. The field strength (*F*) is an index of the strength of elements in the glass matrix introduced by Dietzel.^[^ ^39^ ^]^ It is calculated using Equation 1):

(1) $$F=\frac{Z}{d^{2}}\left(\mathrm{valence}/{Å}^{2}\right)$$

where *Z* represents the valence of the element, and *d* represents the interatomic distance between the element and oxygen ion, which can be calculated as the sum of tshe radii of the element and oxygen ion in Å (= 10^−10^ m). The relative field strength (*F_R_*) was calculated using Equation (2) to compare the properties of glasses:

(2) $$F_{R}=F_{Mg}\left[Mg\right]+F_{Ca}\left[Ca\right]+F_{Zn}\left[Zn\right]+F_{P}\left[P\right]+F_{Si}\left[Si\right]$$

where *F_Mg_*, *F_Ca_*, *F_Zn_*, *F_P_*, and *F_Si_* are 0.53, 0.33, 0.49, 2.10, and 1.57 valence/Å^2^, respectively.^[^ ^5^, ^27^ ^]^ [*Mg*], [*Ca*], [*Zn*], [*P*], and [*Si*] represent the atomic ratios of the glasses calculated from their compositions. *F_R_* values of the glasses are presented in **Table** **1**. X‐ray diffraction (XRD) patterns of the glasses exhibited halo peaks, indicating an amorphous state (Figure S1, Supporting Information).

**Table 1:** Analyzed compositions of the glasses (molar ratio) with standard deviations. The relative field strength (*F_R_*) of the glasses is calculated from the atomic ratio of the composition.

Sample code	Composition / molar ratio (Composition / mol%)					Relative field strength
	MgO	CaO	ZnO	P_2_O_5_	SiO_2_	
SPG‐MC	15.97 ± 0.03 (38.02 ± 0.06)	14.73 ± 0.01 (35.08 ± 0.03)	–	7.60 ± 0.02 (18.09 ± 0.05)	3.70 ± 0.01 (8.82 ± 0.03)	1.029
SPG‐CZ	–	15.54 ± 0.07 (37.00 ± 0.17)	14.90 ± 0.05 (35.48 ± 0.13)	8.05 ± 0.03 (19.46 ± 0.12)	3.50 ± 0.01 (8.34 ± 0.02)	1.034
SPG‐MCZ	10.00 ± 0.02 (23.81 ± 0.05)	10.01 ± 0.03 (23.84 ± 0.08)	10.21 ± 0.03 (24.32 ± 0.08)	8.17 ± 0.05 (19.46 ± 0.12)	3.60 ± 0.02 (8.56 ± 0.04)	1.068
SPG‐MZ	15.84 ± 0.02 (37.71 ± 0.04)	–	14.71 ± 0.03 (35.03 ± 0.06)	7.78 ± 0.05 (18.53 ± 0.12)	3.67 ± 0.01 (8.74 ± 0.02)	1.086

### 2.2 Glass Structure

**Figure 1:**
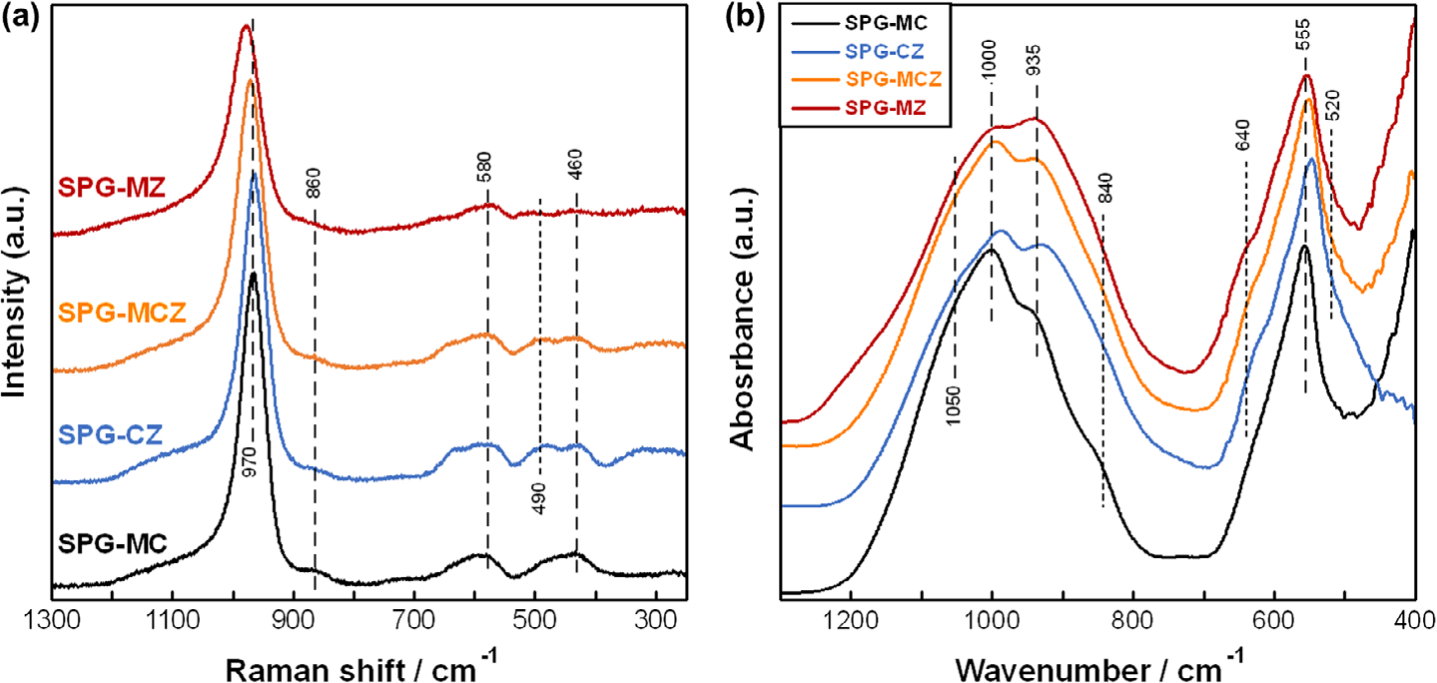
a) Laser Raman and b) FT‐IR spectra of the glasses.

**Figure** **1** shows (a) laser Raman and (b) Fourier transform infrared (FT‐IR) spectra of the glasses. Raman bands corresponding to the orthophosphate (*Q*_P_^0^) group were observed,^[^ ^11^, ^40^, ^41^ ^]^ including the PO_4_ symmetric stretching mode of nonbridging oxygen (970 cm^−1^), the P─O symmetric stretching vibration mode of *Q*_P_^0^ (580 cm^−1^), and the O─P─O bending mode of *Q*_P_^0^ (460 cm^−1^). These glasses may include the PO_3_ symmetric stretching mode of nonbridging oxygen for pyrophosphate (*Q*_P_^1^, observed as a broad band between 1000 and 1200 cm^−1^).^[^ ^10^, ^40^ ^]^ The orthosilicate (*Q*_Si_^0^) group in the glasses was indicated by the band at 860 cm^−1^, corresponding to the symmetric stretching mode of orthosilicate (*Q*_Si_^0^).^[^ ^42^ ^]^ Additionally, bands corresponding to P─O bond local vibrations related to Zn (490 cm^−1^)^[^ ^43^ ^]^ were observed for SPG‐MZ, −MCZ, and ‐CZ, which contain ZnO. FT‐IR bands corresponding to the *Q*_P_^0^ group were also observed, including the symmetric stretching vibration mode of *Q*_P_^0^ (1050 cm^−1^),^[^ ^44^, ^45^ ^]^ the asymmetric stretching vibration mode of *Q*_P_^0^ (1000 cm^−1^),^[^ ^45^, ^46^ ^]^ and the symmetric O─P─O vibration mode (555 cm^−1^).^[^ ^47^ ^]^ The silicate groups were also observed at 840 cm^−1^, corresponding to the Si—O with two nonbridging oxygens per SiO_4_ tetrahedron. The bands at 935 cm^−1^ correspond to Si—O nonbridging oxygen^[^ ^48^ ^]^ and asymmetric stretching of PO_4_^3−^.^[^ ^49^ ^]^ The bands at 640 cm^−1^, which correspond to SPG‐CZ, ‐MCZ, and ‐MZ, are associated with P─O deformation vibration in zinc orthophosphate^[^ ^50^ ^]^ and Zn─O─Si symmetric stretching.^[^ ^51^ ^]^ Glasses containing ZnO exhibited bands corresponding to ZnO_4_ tetrahedra at 520 cm^−1^.^[^ ^47^, ^51^ ^]^ Glasses containing MgO, e.g., SPG‐MC, ‐MCZ, and ‐MZ, exhibited higher absorbance of ~500 cm^−1^, which may correspond to Mg—O vibration.^[^ ^52^ ^]^

**Figure 2:**
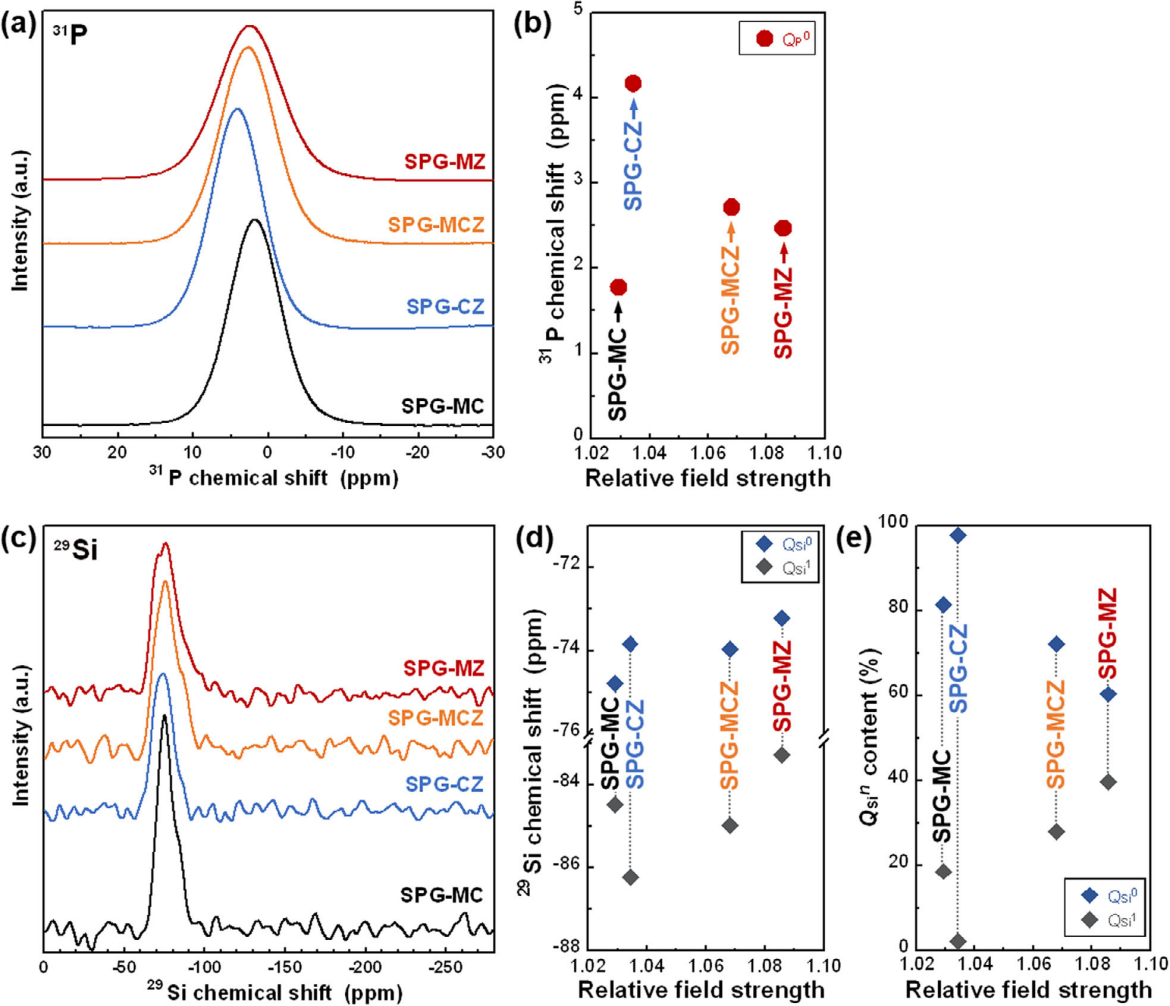
a) ^31^P MAS NMR spectra of the glasses and b) the peak top position of *Q*_P_^0^ plotted against *F_R_*. c) ^29^Si MAS NMR spectra of the glasses. d) Peak top positions of *Q*_Si_^0^ and *Q*_Si_^1^ and e) *Q*_Si_ *^n^* contents in the glasses plotted against *F_R_*.

**Figure** **2** shows (a) ^31^P and (c) ^29^Si magic angle spinning nuclear magnetic resonance (MAS NMR) spectra of the glasses. The ^31^P MAS NMR spectra of the glasses exhibited a sole peak between −10 and 10 ppm, corresponding to the *Q*_P_^0^ group.^[^ ^15^, ^53^ ^]^ Figure 2b shows the peak top position plotted against *F_R_*. The peak top positions of *Q*_P_^0^ were low‐field‐shifted from SPG‐MC to SPG‐CZ, which substituted ZnO for MgO, and high‐field‐shifted with increasing *F_R_*, which substituted MgO for CaO. The ^29^Si MAS NMR spectra of the glasses exhibited a peak between −100 and −50 ppm, as shown in Figure 2c, corresponding to the *Q*_Si_^0^ (≈ −74 ppm) and *Q*_Si_^1^ (≈ −85 ppm) groups.^[^ ^14^, ^54^ ^]^ The spectra were simulated assuming Gaussian lines for the *Q*_Si_^0^ and *Q*_Si_^1^ groups using Igor Pro. The fractured peak top position of *Q*_Si_^0^ shifted to a lower field with increasing *F_R_*. For the *Q*_Si_^1^ peak, the top position shifted from SPC‐MC to SPG‐CZ, which substituted ZnO for MgO. Then, it was low‐field‐shifted with increasing *F_R_*, which substituted MgO for CaO, as shown in Figure 2d. Figure 2e shows the *Q*_Si_ *^n^* (*n*: number of bridging oxygens) contents of the glasses. The *Q*_Si_° content increased from SPG‐MC to SPG‐CZ and then decreased with increasing *F_R_*. In the case of *Q*_Si_^1^, the trend was opposite to that of *Q*_Si_^0^.

**Figure 3:**
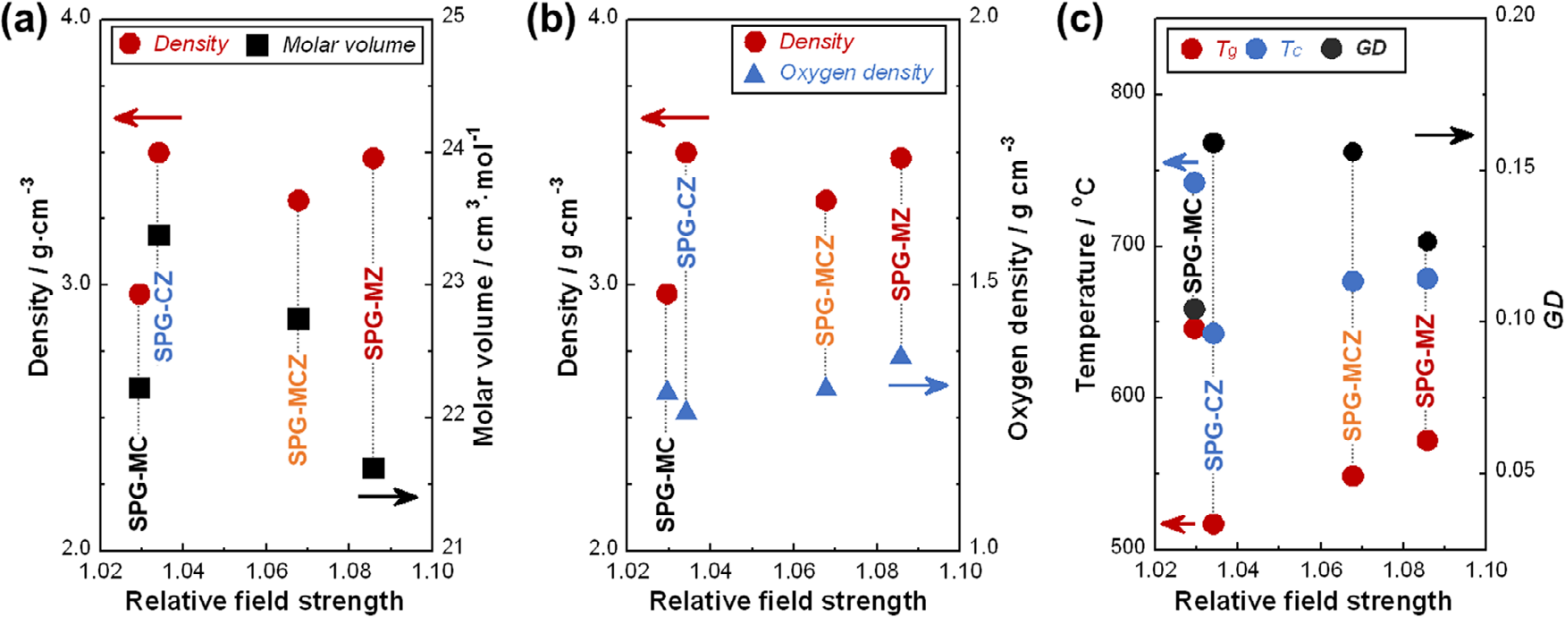
a) Density and molar volume, b) density and oxygen density, and c) glass transition temperature (*T_g_*), crystallization temperature (*T_c_*), and *GD* of the glasses plotted against *F_R_*.

**Figure** **3** shows (a, b) density, molar volume, and oxygen density, and (c) glass transition temperature (*T_g_*), crystallization temperature (*T_c_*), and glassification degree (*GD*) of the glasses. The densities of glasses increased from SPG‐MC to SPG‐CZ, in which ZnO was substituted with MgO. The glasses containing ZnO exhibited no significant differences in density. The molar volume of glasses increased from SPG‐MC to SPG‐CZ, and the values of glasses containing ZnO decreased with an increase in *F_R_*. The oxygen density of glasses decreased from SPG‐MC to SPG‐CZ. The values of glasses containing ZnO increased slightly with an increase in *F_R_*. *T_g_* and *T_c_* of the glasses exhibited similar trends, with the values decreasing from SPC‐MC to SPG‐CZ and then increasing with *F_R_*. *GD* of the glasses exhibited opposite trends to *T_g_* and *T_c_*.

### 2.3 Ion‐Release Behavior of Glasses

**Figure 4:**
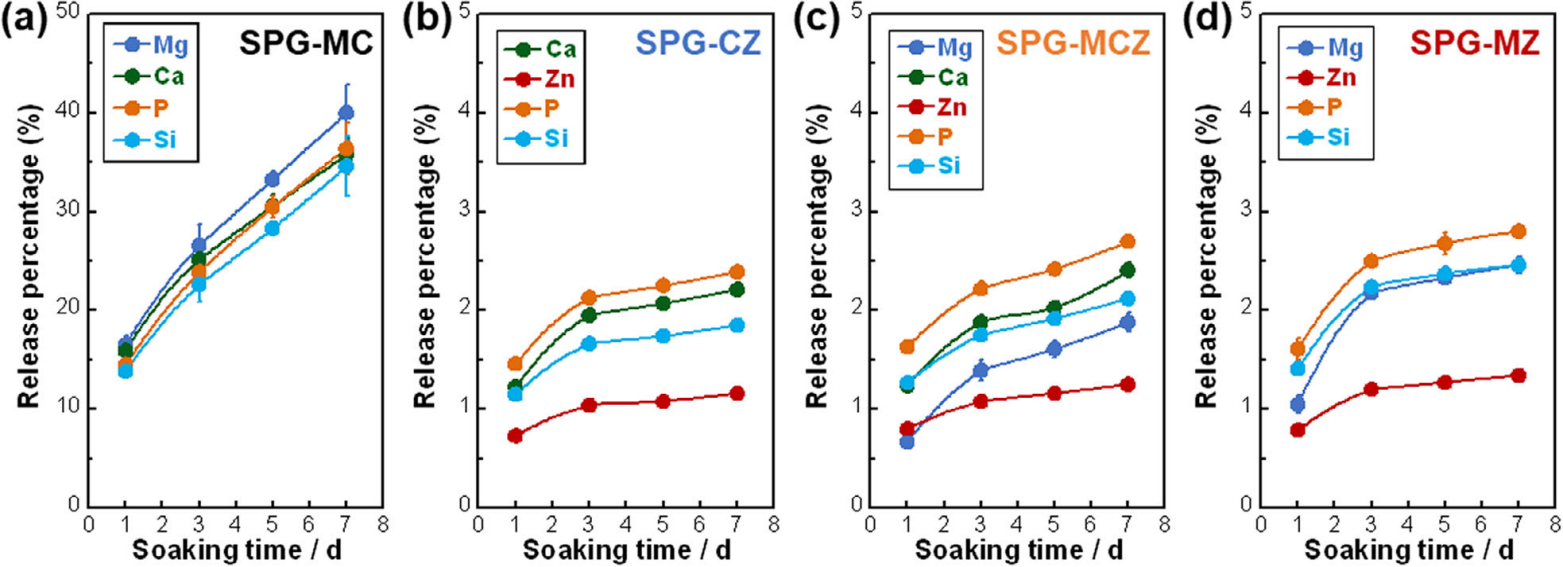
Ion‐release percentages of a) SPG‐MC, b) SPG‐CZ, c) SPG‐MCZ, and d) SPG‐MZ. The error bar represents the standard deviation. Lines are for visual guidance.

**Figure** **4** shows the percentage of ions released from the glasses. The ion‐release percentage of SPG‐MC increased from 15% to 35% from day 1 to day 7 (Figure 4a), whereas the value increased from 1% to 2.5% for the glasses containing ZnO (Figure 4b–d). Thus, the glasses exhibited sustained releasability. On day 7, SPG‐MC exhibited an ion‐release percentage of approximately 35%, whereas glasses containing ZnO exhibited a significantly lower release percentage of 2.5%. The Zn^2+^ ion concentrations of the glasses in TBS are shown in Figure S2 (Supporting Information). The concentration of Zn^2+^ ions in TBS increased in the following order: SPG‐MCZ < SPG‐CZ < SPG‐MZ. XRD patterns of the glasses soaked for 7 d exhibited halo peaks, indicating an amorphous state (Figure S3, Supporting Information).

### 2.4 Antibacterial Activity of Glasses

**Figure 5:**
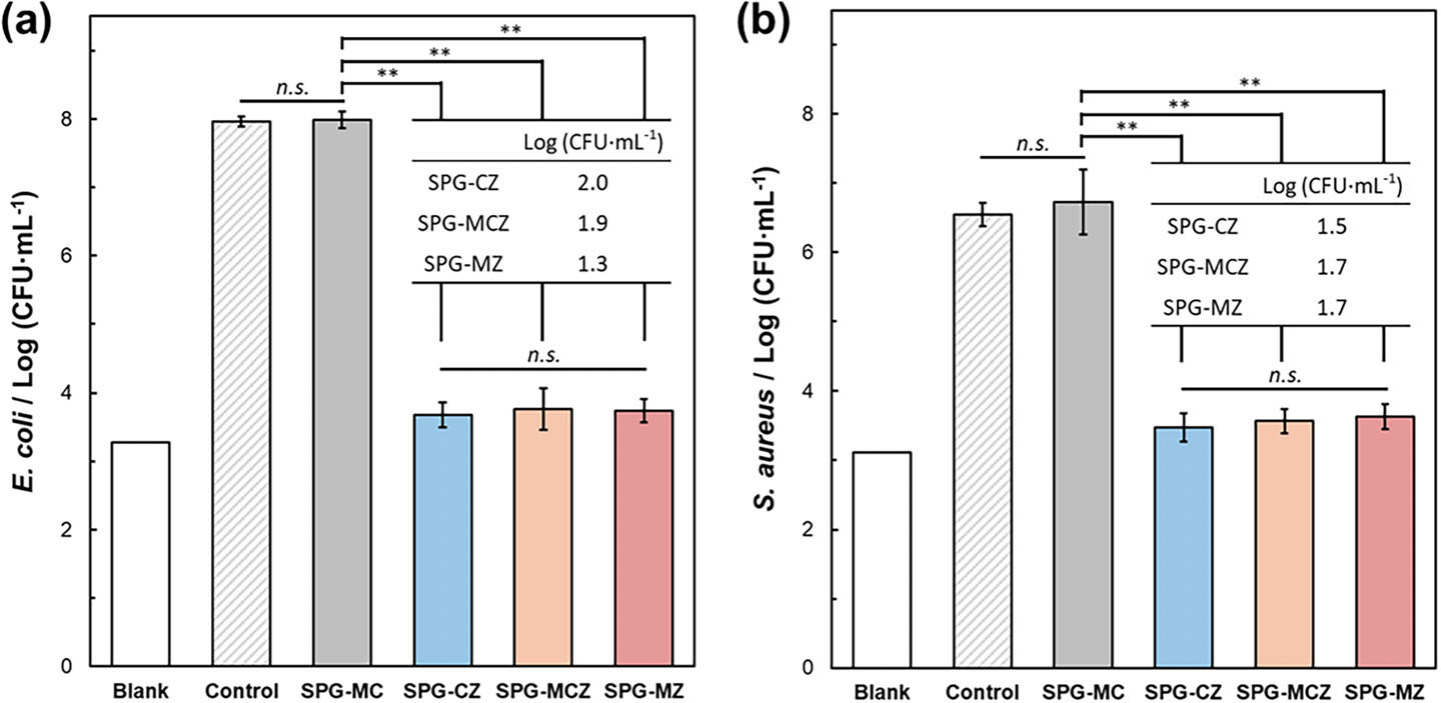
CFU of a) *E. coli* and b) *S. aureus* after cultivation for 24 h with glass granules. The error bar represents the standard deviation. “Blank” indicates the medium without bacteria. The table shows the number of CFU of the bacteria determined via the plate‐count agar method. (^**^: *p* < 0.01 versus SPG‐MC; *n.s*.: not significant).

**Figure** **5** shows the number of colony‐forming units (CFU) of *E. coli* and *S. aureus* after 24 h of cultivation with the glass granules. Here, “Control” indicates the number of CFU after cultivation for 24 h without the addition of glass granules, and “Blank” indicates the values measured by rapisco using the medium without bacteria. SPG‐MC, which did not contain ZnO, exhibited similar values to the control. In contrast, glasses containing ZnO, such as SPG‐CZ, SPG‐MCZ, and SPG‐MZ, exhibited values of ≈ 3, which were similar to the blank values. CFUs of the samples containing ZnO were counted using the platecount agar method. The samples exhibited values of ≈1.7, which were more than two orders of magnitude smaller than those of the control.

### 2.5 Cell Viability and Gene Expression Behavior of Glasses

**Figure 6:**
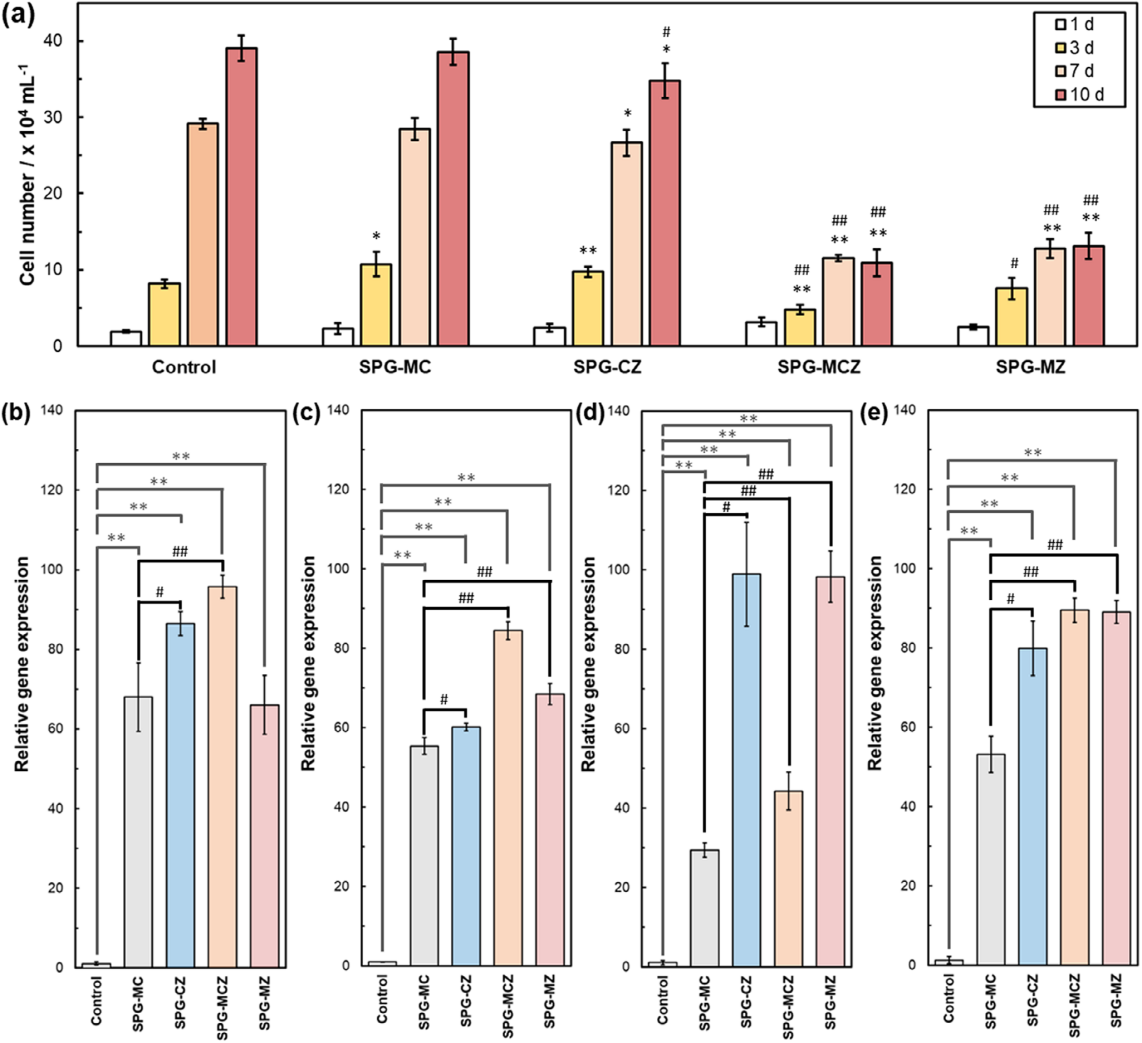
a) Cell numbers after 1–10 d of cultivation with ion‐extraction media prepared with the glass powders. Relative gene expression levels of b) Col I, c) ALP, d) OPN, and e) OCN after 2 weeks of culture with ion‐extraction differential media. The control was cultured in the differential medium. The error bar represents the standard deviation. (^*^: *p* < 0.05 versus Control, ^**^: *p* < 0.01 versus Control, ^#^: *p* < 0.05 versus SPG‐MC, ^##^: *p* < 0.01 versus SPG‐MC for the same culture period).

**Figure** **6**a shows the number of cells cultured in the medium for ion extraction from the glasses. The ion concentrations in the medium are presented in Table S1 (Supporting Information). The cell numbers of SPG‐MC and SPG‐CZ groups were similar to those of the control. However, the cell numbers of SPG‐MCZ and SPG‐MZ were significantly smaller than those of the control and SPG‐MC. The relative gene expression behaviors of the glasses are shown in Figure 6b–d. The gene expression levels of ALP, collagen I (Col I), OPN, and OCN after two weeks of cultivation were significantly upregulated in the ion‐extraction medium compared with the control. The Col I gene expression levels in SPG‐CZ and SPG‐MZ groups were significantly upregulated relative to those in the SPG‐MC group. ALP, OPN, and OCN gene expression were significantly increased for the glasses containing ZnO compared with SPG‐MC, which did not contain ZnO.

## 3 Discussion

### 3.1 Glass Structure and Ion‐Release Behavior

The nominal (**Table** **2**) and analytical (Table 1) compositions of the glasses were similar, indicating that no evaporation or loss occurred during the melt‐quenching process. The amorphous nature of the glasses was confirmed by the XRD halo patterns. In this work, the glasses contained divalent oxides of MgO, CaO, and/or ZnO, and the relative field strengths (*F_R_*, Equation 2) were calculated from the analyzed compositions for comparison between the glasses. The Raman peaks corresponding to the nonbridging oxygen of *Q*_P_^0^ blue‐shifted with an increase in *F_R_* (Figure 1a). The glass network structures of SPGs and PIGs exhibit highly ionic bonding states.^[^ ^3^, ^9^ ^]^ Thus, the peak position of nonbridging oxygen in *Q*_P_^0^ is easily influenced by divalent ions bonding to phosphate groups and undergoes a blue shift due to the increase in the P─O bonding length of *Q*_P_^0[^ ^55^, ^56^ ^]^ caused by cations with higher field strength, as represented by *F_R_*.

**Table 2:** Nominal compositions of the glasses and their sample codes.

Sample code	Composition / molar ratio (Composition / mol%)				
	MgO	CaO	ZnO	P_2_O_5_	SiO_2_
SPG‐MC	15 (35.75)	15 (35.75)	–	8 (19.00)	4 (9.50)
SPG‐CZ	–	15 (35.75)	15 (35.75)	8 (19.00)	4 (9.50)
SPG‐MCZ	10 (23.83)	10 (23.83)	10 (23.83)	8 (19.00)	4 (9.50)
SPG‐MZ	15 (35.75)	–	15 (35.75)	8 (19.00)	4 (9.50)

The glasses containing ZnO exhibited Raman peaks corresponding to the local vibration of P─O bonds related to Zn (490 cm^−1^) and FT‐IR peaks corresponding to the P─O deformation vibration of zinc orthophosphate and symmetric stretching of Zn─O─Si bonds (640 cm^−1^) and ZnO_4_ tetrahedra (520 cm^−1^). The glasses prepared in this work contain ZnO and/or MgO and are classified as intermediate oxides.^[^ ^5^ ^]^ ZnO and MgO in phosphate glasses can enter the phosphate chain structure in the form of ZnO_4_^2−^ and/or MgO_4_^2−^ tetrahedra at high concentrations to form P─O─Zn/Mg bonds.^[^ ^28^, ^57^, ^58^ ^]^ Similarly, Si─O─Zn/Mg bonds are formed in silicate and phosphosilicate glasses containing ZnO or MgO.^[^ ^59^, ^60^ ^]^ In the case of ZnO in phosphosilicate glasses, ZnO prefers to bond with phosphate groups rather than silicate groups and has a stronger preference for phosphate groups than MgO.^[^ ^61^ ^]^ Additionally, ZnO with a higher content in phosphate glasses preferentially bonds to *Q*_P_^0^ groups in the ZnO_4_^2−^ tetrahedra.^[^ ^62^ ^]^ In contrast, MgO in phosphosilicate glasses is distributed in silica‐rich regions in the form of MgO_4_^2–[^ ^63^, ^64^ ^]^ and preferentially bonds with silicate groups.^[^ ^40^ ^]^ In our previous work, MgO in silicophosphate invert glass contributed to glass network formation with four or five fold coordination according to classical molecular dynamics simulations.^[^ ^65^ ^]^ Therefore, ZnO in SPGs functions as NWF, preferentially forming P─O─Zn bonds in the form of ZnO_4_ tetrahedra. Similarly, MgO forms Si─O─Mg bonds in the form of MgO_4_ tetrahedra.

The ^31^P spectra of the glasses exhibited symmetric peaks corresponding to *Q*_P_^0^ (Figure 2a). The peak top positions were low‐field‐shifted from SPG‐MC to SPG‐CZ (Figure 2b), indicating the reduction in electron density. The Mg^2+^ ions in SPG‐MC preferentially bonded to silicates, whereas the Ca^2+^ ions bonded to phosphate groups, as discussed above. In the case of SPG‐CZ, Zn^2+^ ions preferentially bonded to phosphate groups. The electrons belonging to *Q*_P_^0^ groups of SPG‐MC were pulled to the Ca^2+^ side, and those of SPG‐CZ were pulled to the Zn^2+^ side. Thus, the electron density of *Q*_P_^0^ in SPG‐CZ was lower than that in SPG‐MC because the *F* of Zn (0.49) exceeded that of Ca (0.33), and the electrons of *Q*_P_^0^ were pulled to the Zn^2+^ ion side. The peak top positions between SPG‐CZ and SPG‐MZ were high‐field‐shifted with increasing *F_R_*, and the MgO content in the composition gradually increased (Figure 2b). The *F* of Mg is 0.53 or 0.45 with four or six fold coordination, respectively.^[^ ^5^ ^]^ MgO in the SPGs preferentially bonds with silicate groups, forming Si─O─Mg bonds with MgO_4_ tetrahedra, as previously described. MgO substituted for CaO/ZnO can adopt six fold coordination around the *Q*_P_^0^ group, and the electron density of *Q*_P_^0^ increases because the *F* of six fold‐coordinated Mg (0.45) is lower than that of Zn (0.49).

The ^29^Si MAS NMR spectra of the glasses indicated *Q*_Si_^0^ and *Q*_Si_^1^ groups (Figure 2c). However, the Raman and FT‐IR spectra of the glasses exhibited no bands corresponding to *Q*_Si_^1^ groups. In contrast, the *Q*_Si_° content of SPG‐CZ, which did not contain MgO, was ≈98% (Figure 2e). Takada et al. reported that the ^31^P MAS NMR spectra of six‐coordinated Si in silicophosphate glasses bonded to *Q*_P_^2^ groups exhibited ultraphosphate (*Q*_P_^3^) peaks in experiments and simulations.^[^ ^66^ ^]^ Thus, *Q*_Si_^1^ groups observed in SPG‐MC, ‐MCZ, and ‐MZ could be *Q*_Si_^0^ groups bonded to MgO_4_ tetrahedra. The *Q*_Si_^1^ content between SPG‐CZ and SPG‐MZ increased with MgO substitution for CaO/ZnO, forming Si─O─Mg bonds. The top peak positions of *Q*_Si_^1^ exhibited opposite trends to those of *Q*_P_^0^ (Figure 2d). The electron density of *Q*_Si_^0^ in SPG‐CZ was higher than that in SPG‐MC because the *F* of Ca (0.33) was smaller than that of Mg (0.53), and the electrons of *Q*_Si_^0^ were pulled less to the Ca^2+^ ion side than to the Mg^2+^ ion side. The peak top positions between SPG‐CZ and SPG‐MZ were low‐field‐shifted because the MgO content increased and Si─O─Mg bonds were formed.

In the structure of SPGs, phosphate and silicate mainly contained *Q*_P_^0^ and *Q*_Si_^0^ groups, and ZnO and MgO entered the glass network structure as NWFs in the form of ZnO_4_ and MgO_4_ tetrahedra, respectively. These results suggest that there are no P─O─P, Si─O─Si, or P─O─Si bonds and that the glasses do not contain long‐chain structures. The cumulative network‐forming ability of ZnO and MgO in SPGs explains the presence of mainly *Q*_P_^0^ and *Q*_Si_^0^ groups.

The density of SPG‐MC was lower than those of SPG‐CZ, ‐MCZ, and‐MZ (Figure 3a) because the atomic weight of Zn (65.4) exceeds those of Mg (24.3) and Ca (40.1). In contrast, the oxygen density decreased slightly from SPG‐MC to SPG‐CZ because the ionic radius of Zn^2+^ ions (0.060 nm) is slightly larger than that of Mg^2+^ ions (0.057 nm).^[^ ^67^ ^]^ The densities of the glasses containing ZnO exhibited no significant differences among the samples, but the oxygen density increased with *F*_R_. This is because MgO, which functions as NWF in SPGs, has smaller ionic radius than Ca^2+^ ions substituted for CaO.

*T_g_* and *T_c_* decreased from SPG‐MC to SPG‐CZ because the *F* of Zn was smaller than that of Mg (Figure 3b). From SPG‐CZ to SPG‐MZ, the values increased slightly because MgO, which has larger *F* than Ca, was substituted with CaO. The *GD* values of SPGs exceed 0.1, indicating moderate glass‐forming ability,^[^ ^4^ ^]^ even though the glasses do not contain long‐chain structures. The *GD* of SPG‐CZ was higher than that of SPG‐MC. As discussed above, Zn^2+^ and Mg^2+^ ions preferentially bonded to phosphate and silicate groups, respectively. The phosphate group content in SPGs is higher than that of silicate groups, and the *GD* values were more significantly affected by the structure of phosphate groups than by that of silicate groups. Thus, the *GD* value of SPG‐CZ was higher because the *F* value of Zn was larger than that of Ca, which is similar to the reason for the ^31^P MAS NMR peak shift of *Q*_P_^0^. Hence, the reduction in *GD* values from SPG‐CZ to SPG‐MZ can also be explained by the ^31^P MAS NMR peak shift of *Q*_P_^0^. Briefly, the substituted MgO had a six fold coordination structure around the *Q*_P_^0^ group, as discussed above.

The ion‐release percentage of SPG‐MC (≈ 35% on day 7) was significantly lower than that of SPG‐CZ, ‐MCZ, and ‐MZ (≈2.5% on day 7), which contained ZnO (Figure 4). MgO in SPGs preferentially bonds with silicate groups to form Si─O─Mg bonds, as described in the discussion on the structure of SPGs. Si─O─Mg bonds have been reported to weaken the glass network structure^[^ ^58^ ^]^ and easily induce hydrolysis.^[^ ^68^ ^]^ In contrast, ZnO has been reported to improve the chemical durability of silicate, phosphate, and phosphosilicate glasses by entering the glass network structure and forming P/Si─O─Zn bonds in the form of ZnO_4_ tetrahedra.^[^ ^59^, ^60^, ^61^, ^69^, ^70^, ^71^ ^]^ Thus, the improvement in chemical durability of ZnO‐containing glasses originates from the formation of P/Si─O─Zn bonds in the glass network structure. The Zn^2+^ ion concentration in TBS of SPG‐MCZ was lower than that for SPG‐CZ and ‐MZ, owing to the lower ZnO content. Among the samples, Zn^2+^ ion concentration in TBS was highest for SPG‐MZ (Figure S2, Supporting Information). MgO in PIGs also weakens the glass network structure and easily induces hydrolysis,^[^ ^10^ ^]^ similar to the silicate glasses discussed above. Thus, SPG‐MZ had lower chemical durability than SPG‐CZ and exhibited higher Zn^2+^ ion concentration in TBS. The 50% inhibitory concentration (IC_50_) of Zn^2+^ ions for murine osteoblast (MC3T3‐E1) and fibroblast (L929) cells is 0.09 mM.^[^ ^72^ ^]^ ZnO‐containing glasses release <0.08 mM Zn^2+^ ions and are not expected to be cytotoxic.

### 3.2 Bifunctionality of Glasses for Biomedical Applications

The antibacterial activity of the glasses was evaluated using *E. coli* and *S. aureus*. In this work, the shaking method was used to evaluate the effect of ZnO on glass composition. The conventional shaking method (Society of Industrial Technology for Antimicrobial Articles) uses 1/500 nutrient broth (NB) medium^[^ ^29^ ^]^; however, this medium is not sufficient to increase the bacterial number after 24 h of incubation. In this work, 1/100 NB medium was used, and CFUs of the bacteria increased by four orders of magnitude, which was sufficient to evaluate the antibacterial activity of the glasses (Figure 5). The ZnO‐containing glasses exhibited antibacterial activity and had CFU values > 5 orders of magnitude smaller than those of the control for *E. coli* and *S. aureus*. In contrast, SPG‐MC did not exhibit antibacterial activity. These data indicate that ZnO‐containing SPGs exhibit antibacterial activity by releasing Zn^2+^ ions.

The cell numbers in control, SPG‐MC, and SPG‐CZ were similar, whereas SPG‐MCZ and SPG‐MZ exhibited smaller values (Figure 6 (a)). Zn^2+^ ions exhibit mild cytotoxicity in healthy tissues.^[^ ^72^ ^]^ ZnO‐containing phosphosilicate and phosphate glasses negatively affect human osteoblast (MG63) cells.^[^ ^70^, ^73^, ^74^ ^]^ In contrast, ZnO‐containing PIGs and metaphosphate glasses with controlled Zn^2+^ ion‐release behavior do not show cytotoxic effects or improve cell proliferation.^[^ ^69^, ^75^ ^]^ Additionally, Zn^2+^ ions exhibited cytotoxicity at different concentrations in different cell types; e.g., MC3T3‐E1 cells showed no cytotoxicity, whereas primary osteoblasts cultured with the same samples showed cytotoxicity.^[^ ^76^ ^]^ The Zn^2+^ ion concentrations in the ion‐extraction medium of SPG‐CZ were lower than those for SPG‐MCZ and SPG‐MZ, whereas those for SPG‐MCZ and SPG‐MZ were similar (Table S1, Supporting Information). The Zn^2+^ ion concentrations of SPG‐MCZ and SPG‐MZ in TBS and the culture medium were (≈ 0.07 and 0.06 mM, respectively (Figure S2, Supporting Information). These values were lower than the IC_50_ (≈ 0.09 mM) for MC3T3‐E1 and L929 cells. However, Saos‐2 cells cultured with SPG‐MCZ and SPG‐MZ exhibited smaller numbers on days 3–10, possibly because the Zn^2+^ ions released from the glass had a mild inhibitory effect on proliferation.

The osteogenic markers Col I, ALP, OPN, and OCN were upregulated when cultivated in SPG ion‐extracted media compared with the control. The release of inorganic ions, such as silicate, phosphate, Mg^2+^, Ca^2+^, and Zn^2+^, from SPGs upregulates the osteogenic markers of osteoblasts and is expected to stimulate bone regeneration. The expression of Col I, ALP, OPN, and OCN can be enhanced by silicate^[^ ^21^, ^77^, ^78^, ^79^ ^]^ and phosphate ions.^[^ ^80^, ^81^, ^82^ ^]^ Thus, the upregulation effect of SPGs releases silicate and phosphate ions from the glasses. The expression levels of ALP, OPN, and OCN in ZnO‐containing SPGs were significantly higher than those in SPG‐MC, as Zn^2+^ ions enhance the expression of these genes.^[^ ^34^, ^35^, ^36^ ^]^ Recently, Moriishi et al. reported that OCN—a non‐collagenous protein in bones—is necessary for bone quality and strength, as it adjusts the *c*‐axis orientation of biological apatite (BAp) parallel to collagen fibrils.^[^ ^83^ ^]^ The degree of BAp *c*‐axis orientation correlates well with the Young's modulus of bone tissue and can be used as the main indicator of bone quality.^[^ ^84^ ^]^ Additionally, the recovery of BAp *c*‐axis orientation during bone regeneration is significantly delayed compared with the formation of bone mineral density (BMD, bone quantity).^[^ ^84^ ^]^ Thus, bone quality is the predominant mechanical property of bone tissue, rather than bone quantity.^[^ ^84^, ^85^ ^]^ In our previous work, ZnO‐containing SPGs exhibited higher bone quality than the control (cultured medium without ion extraction from the glass), which is attributed to upregulation of OCN due to the release of Zn^2+^ ions from the glass.^[^ ^76^ ^]^

ZnO‐containing SPGs exhibit antibacterial activity and stimulatory effects on osteogenic markers. These glasses are expected to exhibit bifunctional properties for biomedical applications. Furthermore, the expression level of OCN, which is related to the formation of high‐quality bone, is significantly upregulated by Zn^2+^ in the glasses. Hence, ZnO‐containing SPGs are expected to simultaneously restore bone quantity and quality by releasing therapeutic ions.

## 4 Conclusion

Novel SPGs containing ZnO were prepared as bifunctional biomaterials with enhanced bone formation and antibacterial activity. The glasses were mainly composed of the orthotetrahedral structures of PO_4_^3−^, SiO_4_^4−^, MgO_4_^2−^, and ZnO_4_^2−^. The intermediate MgO and ZnO oxides in SPGs functioned as NWFs and entered the glass network structure by bridging the phosphate and/or silicate groups. Thus, SPGs do not contain P─O─P, Si─O─Si, or P─O─Si bonds, indicating that they comprise a unique glass network structure without long‐chain structures. The chemical durability of ZnO‐containing SPGs was superior to that of SPG‐MC because ZnO acts as NWF to form P/Si─O─Zn bonds. The amount of Zn^2+^ ions released from the ZnO‐containing SPGs was controlled to be <0.09 mM, indicating cytotoxicity to cells. The ZnO‐containing SPGs exhibited excellent antibacterial activity, which was > 5 orders of magnitude lower than that of the control and SPG‐MC. SPG‐MCZ and SPG‐MZ had mild inhibitory effects on cell proliferation induced by Zn^2+^ ions. However, ZnO‐containing SPGs exhibited significant upregulation of osteogenic markers, such as Col I, ALP, OPN, and OCN, compared with the control, owing to the release of inorganic ions from the glasses. The ZnO‐containing SPGs prepared in this work exhibited bifunctional properties suitable for biomedical applications. They are promising bioadaptive materials for controlling gene expression through the release of therapeutic ions.

## 5 Experimental Section

### Preparation of MgO‐CaO‐ZnO‐P_2_O_5_‐SiO_2_ Glasses

ZnO‐containing silicophosphate glasses with nominal compositions were prepared, as listed in Table 2. Glass batches were prepared using MgO (99.0%), CaCO_3_ (99.5%), ZnO (99.5%), H_3_PO_4_ (85% liquid), and SiO_2_ (99.0%). All the reagents were purchased from Kishida Chemical Co. The reagents were manually mixed with distilled water to form a slurry, which was dried at 140 °C overnight. Subsequently, the resulting powders were melted in the Pt crucible at 1500 °C for 30 min, and the melts were quenched by pressing two stainless‐steel plates. The glass powder (2.5 mg) was dissolved in 10 mL of 1 M HNO_3_, and inductively coupled plasma atomic emission spectroscopy (ICP‐AES, ICPS‐8000, Shimadzu) was performed to determine the composition of the glass. The glass nature was confirmed via X‐ray diffractometry (SmartLab SE/B1, Rigaku) using a Cu Kα X‐ray source at 1° min^−1^ with a scan step 0.01°.

### Structural Analysis of Glasses

The glass structure was analyzed using laser Raman spectroscopy in the range of 300–1300 cm^−1^ (NRS‐5100, 532.02 nm, JASCO) and FT‐IR spectroscopy with attenuated total reflectance (FT/IR‐4700, JASCO). The solid‐state ^31^P and ^29^Si MAS NMR (JNM‐ECS400, JEOL) spectra of the glasses were evaluated using a 4.0‐mm rotor. ^31^P MAS NMR (resonance frequency: 161.83 MHz) spectra were acquired with a pulse width of 5.0 µs, recycle delay of 5 s, cumulative number of 256 scans, and spinning speed of 10 kHz. Ammonium dihydrogen phosphate (NH_4_H_2_PO_4_) was used as a reference for the chemical shift with the value of 1.0 ppm. ^29^Si MAS NMR (resonance frequency: 79.43 MHz) spectra were acquired with a pulse width of 5.5 µs, recycle delay of 240 s, cumulative number of 1080 scans, and spinning speed of 6 kHz. 3‐(Trimethylsilyl)‐1‐propaneulfonic acid sodium salt (1.534 ppm) was used as a reference for the chemical shift.

The density of glasses (*ρ_glass_*) was measured using the He pycnometer (AccuPyc II 1340, Shimadzu). The oxygen density (*ρ_oxygen_*) was calculated using Equation (3)^[^ ^86^, ^87^ ^]^:

(3) $$\rho_{oxygen}=\frac{M\left(O\right)\times \left\{\left[MgO\right]+\left[CaO\right]+\left[ZnO\right]+5\left[P_{2}O_{5}\right]+2\left[SiO_{2}\right]\right\}}{M\left(glass\right)/\rho_{glass}}\;$$

where *M*(*O*) and *M*(*glass*) represent the atomic weight of oxygen (16) and the molar weight of glasses, respectively, and [*MgO*], [*CaO*], [*ZnO*], [*P_2_O_5_*], and [*SiO_2_*] represent the molar fractions of components. The thermal behavior of glass was examined using differential thermal analysis (DTA, Thermo Plus TG8120, Rigaku). The glass transition (*T*_g_) and crystallization (*T*_c_) temperatures were determined using the DTA traces. The glassification degree (*GD*), which is an indicator of glass‐forming ability, was calculated using Equation (4)^[^ ^4^, ^88^ ^]^:

(4) $$GD=\frac{T_{c}-\;T_{g}}{T_{g}}\;\left[\frac{K}{K}\right]$$

### Ion‐Release Behavior of Glasses

Glass granules with sizes of 125–250 µm were used for evaluating the ion‐release behavior. 50 mM of TBS was prepared by dissolving 6.118 g of tris(hydroxymethyl)aminomethane (NH_2_C(CH_2_OH)_3_, Kishida Chemical Co.) in 1 L of distilled water and adjusting the pH to 7.4 with 1 m HCl at 37 °C. Three containers were prepared for each time point (1, 3, 5, 7 d) with 15 mg of glass granules in 15 mL of TBS. The ion‐release behavior of glasses was analyzed over 7 d. The concentrations of Ca^2+^, Mg^2+^, Zn^2+^, phosphate, and silicate ions in TBS were measured using ICP‐AES. The molar release percentage of each ion was determined using Equation (5)^[^ ^89^, ^90^ ^]^:

(5) $$Release\;precentage\;\left(\%\right)=\frac{\left(C_{ion}/M_{w.atom}\right)\;\times \;10^{5}}{\left(Frac_{mol}\;\times \;M_{w.glass}\right)/\left(m_{glass}\;\times \;V_{solution}\right)},$$

where *C_ion_* (mg L^−1^) represents the concentration of the element of interest, *M*_*w*.*atom*_ (g) represents the atomic weight of an element, *m_glass_* (g) represents the mass of the sample added to TBS, and *V_solution_* (L) represents the volume of TBS. *Frac_mol_* represents the molar fraction of the element, and *M*_*w*.*glass*_ (g) represents the molar weight determined from the glass‐composition measurement results. After soaking for 7 d, the glass granules were analyzed using XRD with a scan step of 0.05° at 0.1° min^−1^.

### Antibacterial Activity of Glasses

The antibacterial activity of the glasses was evaluated using the modified shaking method (Society of Industrial Technology for Antimicrobial Articles).^[^ ^29^, ^45^ ^]^ Gram‐negative (*E. coli*, strain NBRC3972) and gram‐positive (*S. aureus*, strain NBRC12732) bacteria were used in this work. The NB medium was prepared by dissolving 5 g of beef extract (Nacalai Tesque), 10 g of meat peptone (Nacalai Tesque), and 5 g of sodium chloride (Fujifilm Wako Pure Chemical) in 1 L of distilled water, followed by autoclaving sterilization at 121 °C for 20 min. Glass granules with sizes of 125–250 µm were dry‐sterilized at 180 °C for 90 min. 1 mg of glass granules was soaked in 1 mL of 1/100 NB medium with an initial concentration of 4 × 10^4^ CFU mL^−1^ *E. coli* or *S. aureus* in 12‐well plates (*n* = 4). In this work, a five fold higher concentration of NB medium was used compared with the ordinary shaking method (1/500 NB medium). The media were subsequently cultured for 24 h at 37 °C in the orbital‐shaking incubator (90 rpm). After cultivation, the colonies were counted using rapisco (RF‐mk2; Yamato Scientific Co.) and microbial fluorescent staining. Microbial fluorescence staining was performed according to the manufacturer's instructions. Briefly, 300 µL of the culture medium incubated with glass granules was filtered through a 0.4‐µm membrane filter, and the bacteria were captured on the filter. The filter was washed with 1 mL of staining buffer solution (SBS). Subsequently, 200 µL of carboxyfluorescein diacetate (CFDA, DOJINDO Laboratories) staining solution was added to the filter, followed by incubation at 37 °C for 5 min under dark conditions. After staining, the filter was washed with 1 mL of SBS and measured using rapisco. The SBS was prepared with 40 g of sodium chloride (Fujifilm Wako Pure Chemical), 1 g of potassium chloride, 5.75 g of disodium hydrogen phosphate (Fujifilm Wako Pure Chemical), and 1 g of potassium dihydrogen phosphate (Fujifilm Wako Pure Chemical) in 1 L of distilled water with 0.5 mM ethylenediaminetetraacetic acid (EDTA, Fujifilm Wako Pure Chemical) and then filter‐sterilized. The glasses containing ZnO exhibited similar values to the blank sample (bacteria‐free medium), and the number of colonies was determined using the plate‐count agar method (*n* = 1). The agar plates were prepared via autoclave sterilization at 121 °C for 20 min using NB with 1.5% agar (Nacalai Tesque) and poured into plastic Petri dishes.

### Cell Viability and Gene Expression Behavior of Glasses

Human osteoblast‐like cells (Saos‐2, TKG0469, Cell Resource Center for Biomedical Research, Institute of Development, Aging, and Cancer, Tohoku University) were used to test the cell viability and gene expression behavior of the glasses. Saos‐2 cells were cultured in McCoy's 5A medium (Cytiva) containing 15% fetal bovine serum (FBS; Nichirei) and 1% penicillin‐streptomycin solution (Fujifilm Wako Pure Chemical) at 37 °C under 5% CO_2_. The glass powders were dry‐sterilized at 180 °C for 90 min and then soaked in McCoy's 5A medium (1 mg mL^−1^) for 24 h at 37 °C. Subsequently, the media were filtered (0.22 µm filter) to remove the glass powders. The ion concentrations of the ion‐extraction medium from the glass powders were measured via ICP optical emission spectrometry (ICP‐OES; Avio500, PerkinElmer). For the cell‐viability test for the ion‐extraction media from the glasses, Saos‐2 cells were seeded into 96‐well plates by adding 100 µL of ion‐extraction media with a cell concentration of 2.0 × 10^4^ cells mL^−1^ (*n* = 3). Cells cultured in McCoy's 5A medium were used as controls. The ion‐extraction medium was replaced after 1, 3, and 7 d of culture. After the preset culture time, the cells were washed twice with McCoy's 5A medium without phenol red (Cytiva). Subsequently, the medium without phenol red was replenished, followed by the addition of 10 µL of PrestoBlue Cell Viability Reagent (Invitrogen) and 1 h of incubation. The resulting media (100 µL) were transferred to a new 96‐well plate and measured with 535 nm fluorescence excitation and fluorescence at 612 nm using the microplate reader (Infinite F200 PRO, Tecan). The number of cells was calculated from the standard curve of cell number versus fluorescence emission intensity.

The gene expression behavior of Saos‐2 cells cultured in the ion‐extraction media was evaluated (*n* = 3). The cells were seeded into 12‐well plates by adding 1 mL of McCoy's 5A medium with 15% FBS and 1% penicillin‐streptomycin solution at a concentration of 5.0 × 10^4^ cells mL^−1^. After 1 d of culture, the medium was replaced with differential medium containing the ions extracted from the glasses. The differential medium was adjusted to the final concentration of 50 µg mL^−1^ ascorbic acid (Nacalai Tesque), 10 mM β‐glycerophosphate (TCI Chemicals), and 50 nM dexamethasone (Fujifilm Wako Pure Chemical).^[^ ^76^, ^91^ ^]^ The control was cultured in the differential medium without ion extraction from the glass. The medium was replaced twice per week after one day of culture. After two weeks of cultivation, the cells were treated with TRIzol Reagent (Invitrogen). Total ribonucleic acid (RNA) was isolated from cells according to the manufacturer's instructions. Complementary deoxyribonucleic acid (cDNA) was synthesized from the isolated RNA using the PrimeScript RT Master Mix (Takara Bio). Subsequently, the cDNA, primers, and SYBR green mix (iTaq Universal SYBR Green Supermix; Bio‐Rad) were mixed according to the manufacturer's instructions for real‐time polymerase chain reaction (PCR; CFX96; Bio‐Rad) analysis. Primers for glyceraldehyde 3‐phosphate dehydrogenase (GAPDH, Takara Bio), collagen type I (Col I, Takara Bio), ALP (Takara Bio), OPN (Hokkaido System Science), and OCN (Hokkaido System Science) were used in this work (**Table** **3**). Gene expression levels were evaluated using the ΔΔCt method with GAPDH as the housekeeping gene. The two means were compared using a two‐tailed unpaired Student's *t*‐test, followed by an *F*‐test for homoscedasticity.

**Table 3:** Sequence of primers used in this work.

Primer		Sequence
GAPDH	Forward	GCACCGTCAAGGCTGAGAAC
	Reverse	TGGTGAAGACGCCAGTGGA
Col I	Forward	CCCGGGTTTCAGAGACAACTTC
	Reverse	TCCACATGCTTTATTCCAGCAATC
ALP	Forward	GGACCATTCCCACGTCTTCA
	Reverse	CAGGCCCATTGCCATACA
OPN	Forward	ATCTCCTAGCCCCACAGAAT
	Reverse	CATCAGACTGGTGAGAATCATC
OCN	Forward	GACTGTGACGAGTTGGCTGA
	Reverse	CTGGAGAGGAGCAGAACTGG

## Conflict of Interest

The authors declare no conflict of interest.

## Supporting information



Supporting Information

## Data Availability

The data that support the findings of this study are available from the corresponding author upon reasonable request.
