# Supplementary material for: Preparation of Bifunctional Orthosilicophosphate MgO‐CaO‐ZnO‐P2O5‐SiO2 Glasses: In Vitro Evaluation of Antibacterial Activity and Osteoblast Gene Expression Behavior
Source: Adv Healthc Mater. 2025 Sep 6;15(1):e02546. doi: 10.1002/adhm.202502546 (PMC12790307; doi:10.1002/adhm.202502546)
Supplement: Supplementary file 1 — Supporting Information [file ADHM-15-0-s001.docx]

Supporting Information

Preparation of Bifunctional Orthosilicophosphate MgO-CaO-ZnO-P_2_O_5_-SiO_2_ Glasses:
*in vitro* Evaluation of Antibacterial Activity and Osteoblast Gene Expression Behavior

Sungho Lee*, Hayato Asano, Makoto Sakurai, Takayoshi Nakano, and Toshihiro Kasuga

**Figure S1.** XRD patterns of the glasses.

**Table S1.** Ions concentrations of ion**-**extraction media. **“**Control**”** represents McCoy’s 5A medium with 15% FBS and 1% penicillin-streptomycin solution.

| **Sample**  **code** | **Ion concentration / mg·L^-1^** | | | | |
| --- | --- | --- | --- | --- | --- |
|  | **Mg** | **Ca** | **Zn** | **P** | **Si** |
| Control | 17.6 | 31.0 | - | 119.3 | - |
| SPG-MC | 23.0 | 41.3 | - | 123.6 | 2.2 |
| SPG-CZ | 17.3 | 40.5 | 0.9 | 121.0 | 0.2 |
| SPG-MCZ | 18.8 | 38.9 | 3.5 | 117.8 | 1.0 |
| SPG-MZ | 19.9 | 35.8 | 4.2 | 119.4 | 1.0 |

**Figure S2.** Zn^2+^ ion concentration in TBS from the glasses. The error bar represents the standard deviation. Lines are for visual guidance.

**Figure S3.** XRD patterns of the glasses after soaking for 7 d.
